# Experiences of People Diagnosed with High Levels of LDL Cholesterol and Atherosclerotic Cardiovascular Disease: Results from a Multinational Qualitative Study

**DOI:** 10.5334/gh.1441

**Published:** 2025-07-15

**Authors:** Neil Johnson, Joe Vandigo, Fernanda de Carvalho, Celina Gorre, Tanya Hall, Susan E. Hennessy, Dhruv S. Kazi, Kornelia Kotseva, Patsy Petrie, David Kelly, Ankita Saxena, Elisabeth M. Oehrlein

**Affiliations:** 1 Global Heart Hub, Galway, Connacht, Ireland; 2 Applied Patient Experience, Washington, DC, United States of America; 3 Instituto Lado a Lado pela Vida, São Paulo, São Paulo, Brazil; 4 WomenHeart, Washington, DC, United States of America; 5 Hearts4heart, Perth, Western Australia, Australia; 6 University of California, San Francisco, San Francisco, CA, United States of America; 7 Richard A. and Susan F. Smith Centre for Outcomes Research, Cardiac Critical Care Unit, Beth Israel Deaconess Medical Centre, Harvard Medical School, Boston, MA, United States of America; 8 National Institute for Prevention and Cardiovascular Health, University of Galway, Ireland; 9 Imperial College Healthcare NHS Trust, London, United Kingdom; 10 HEART UK & FH Europe Foundation Ambassador, Linlithgow, United Kingdom

**Keywords:** Cardiovascular risk, qualitative study, patient experience, diagnostic pathways, lifestyle changes

## Abstract

**Background:**

Elevated low-density lipoprotein cholesterol (LDL-C) levels are a leading risk factor for atherosclerotic cardiovascular disease (ASCVD), a major global cause of illness and death. Patients’ qualitative insights about experiences, priorities, and needs are essential for creating more targeted, patient-centered quality improvement interventions.

**Objectives:**

To document the experiences of people with high levels of low-density LDL-C in three countries.

**Methods:**

Qualitative study of 60-min in-depth interviews with 50 adult patients from Australia, Brazil, and the United States. The study was overseen by a Steering Committee comprising patients, patient advocates, researchers, and cardiologists. The interviews explored pathways and barriers to high LDL-C diagnosis; the burden of managing high LDL-C and the awareness of the association between high LDL-C and cardiovascular risks. The data were analyzed by applying a structured, team-based approach to coding qualitative data.

**Results:**

There were three main pathways to diagnosing high cholesterol: routine physical exams conducted by primary care providers; symptomatic presentations or incidental findings during emergency visits and through a healthcare visit for another condition, frequently diabetes. Healthcare providers’ communication styles influenced patients’ perceptions of their conditions. Two-thirds of participants (*n* = 33) attempted lifestyle changes after their high cholesterol diagnosis, but work schedules and daily routines posed barriers to maintaining healthy habits. Some participants who experienced ASCVD events waited hours or days before seeking care, assuming their symptoms were not serious. After diagnosis of an ASCVD event, many patients feared death and worried about their families’ futures. When asked about potential improvements to their current therapy, 21 patients mentioned reduced administration frequency.

**Conclusions:**

This pilot study provides insights into patients’ experiences living with and managing elevated LDL-C. It describes opportunities for policymakers and healthcare providers to improve the detection of elevated LDL-C and support patients in understanding risks and strategies for reducing the risk of ASCVD events.

## Introduction

Atherosclerotic cardiovascular disease (ASCVD) is a major global cause of illness and death, with its prevalence nearly doubling over the past 30 years (1). In addition to the impact on patient outcomes, cardiovascular disease places a major economic burden on countries, both with respect to healthcare costs and productivity losses (2,3,4). ASCVD encompasses a range of conditions, including acute coronary syndrome, myocardial infarction, stable or unstable angina, coronary or other arterial revascularization, stroke, transient ischemic attack (TIA), and peripheral arterial disease (PAD), including aortic aneurysm.

Clinical guidelines emphasize the importance of adopting healthy lifestyles and addressing modifiable risk factors for ASCVD, such as elevated low-density lipoprotein cholesterol (LDL-C), smoking, hypertension, and dysglycemia. Other causes of high cholesterol are non-modifiable and may not be able to be controlled through lifestyle adjustments or behavior changes alone. For example, certain genetic conditions, including Familial Hypercholesterolemia (FH) and lipoprotein(a) or Lp(a) can put people at greater risk for high cholesterol and ASCVD (5,6). Lowering LDL-C levels effectively reduces ASCVD risk, especially in high-risk populations (7). Effective LDL-C-lowering therapies include statins, non-statin medications (e.g., ezetimibe), and combination treatments (e.g., bile acid sequestrants and ezetimibe or PCSK9 inhibitors added to statin therapy).

However, despite the availability of these therapies, many patients fail to achieve LDL-C targets. A recent European observational study revealed that 80% of high- and very high-risk patients did not meet the 2019 European Society of Cardiology/European Atherosclerosis Society (ESC/EAS) LDL-C guidelines (8). In the United States (US), a study estimated that in 2019, 42% of Americans with ASCVD who were prescribed a lipid-lowering therapy did not fill their prescription in the previous 5 years, and the majority of ASCVD patients were not at their target LDL-C goal (9). In a Brazilian population of people at risk for cardiac events, less than 3% reported receiving statins (10). Over the past two decades, EUROASPIRE surveys have consistently reported poor lifestyle management, high rates of smoking, obesity, central obesity, and diabetes, as well as inadequate control of blood pressure, lipids, and glucose in people with established coronary heart disease (CHD) and those at high risk for cardiovascular disease (11,12).

Despite the critical public health need to address barriers to achieving LDL-C targets and promoting healthy lifestyles, few studies explore the experiences, perspectives, and goals of patients and their families regarding ASCVD management and prevention. Past qualitative studies focus on collecting patient feedback on the effectiveness of specific interventions, such as a lifestyle counseling program, shared decision-making, or information seeking (13,14,15).

Even qualitative research about barriers to adopting LDL-C and ASCVD care plans often stems from physicians. For example, in a recent study in Singapore, physicians identified the following barriers: patients’ lack of knowledge and awareness, patients’ fear of side effects, negative external influences on patients, poor doctor–patient relationship, time constraints during consultations, physicians’ unfamiliarity with guidelines, low health literacy among the local population and lack of national policy (16). A physician survey identified key barriers to achieving these goals in post-myocardial infarction (MI) patients, including poor adherence to lipid-lowering therapies (LLT), side effects from LLT, and insufficient patient education about LDL-C management and lifestyle changes (17).

Qualitative insights directly from patients about their experiences, priorities, and needs regarding LDL-C and ASCVD are essential for creating more targeted, patient-centered interventions. Addressing this gap is especially important given the increasing emphasis on involving patients as active partners in healthcare research and delivery (18,19). This patient-group-led pilot study aimed to address this gap by documenting the experiences of people with high cholesterol and ASCVD in three countries with diverse healthcare systems: Australia, Brazil, and the United States. Specifically, this work explored diagnostic pathways, barriers, and facilitators to LDL-C management adherence and insights to guide future drug development using a patient-centered approach.

## Methods

### Steering committee

The study was guided by a seven-member Steering Committee comprising a patient, patient advocates, researchers, and cardiologists. The Steering Committee was convened to guide the direction and goals of this research study. All are co-authors of this publication and are listed in Supplementary Appendix A. The committee co-created the study protocol, interview guide, and screening criteria during the project kick-off meeting and subsequent ad hoc engagements and email communications. Specifically, the committee guided the topics and discussion questions to address in the interview guide, provided input on the screener and demographic information collected, and contributed to the cultural adaptation of the interview guide. The Steering Committee was convened formally twice during the project to review and interpret preliminary findings and plan dissemination. Individual consultation calls were also conducted with committee members to refine the study approach and conclusions.

### Interviews

This qualitative study involved interviews with adult patients from Australia, Brazil, and the United States. The interview guide was adapted from the National Health Council (NHC)’s Patient Experience Mapping Toolbox (20) (PEMT) by the study team and, through discussions with the Steering Committee, explored:

1. Pathways and barriers to high LDL-C diagnosis and ASCVD events, including awareness of the association between high LDL-C and cardiovascular risks
2. The burden of managing high LDL-C
3. Perspectives on unmet treatment needs and outcomes

Interviews, conducted virtually using Zoom™ between November 2023 and March 2024, lasted up to 60 min. Participants chose whether to appear on camera. Moderators, native speakers of the interviewees’ primary language, conducted all interviews, which were audio-recorded and translated/transcribed into English for analysis. The full interview guide is available in Supplementary Appendix B.

### Eligibility

Patients diagnosed with high LDL-C by a physician at least 2 years prior were eligible for inclusion. Proof of diagnosis was obtained through a physician’s note, a copy of a medical record showing diagnosis, or a current prescription for an LDL-C medicine. The research team sought to balance between people previously hospitalized for an ASCVD event (e.g., heart attack, unstable angina, ischemic stroke, and peripheral artery disease) at least 1 year prior and those who were not. Participants were recruited via social media, referrals from healthcare providers, market research databases, and patient advocacy organizations. Recruitment targets were based on empirical research regarding when theme saturation from interviews is typically achieved and thus targeted at a minimum of 15 people per country, for 45 participants. (21) An equal distribution between men and women at the overall study level was also sought.

### Data analysis

We adapted Giesen and Roeser’s recommendations for structuring a team-based approach to coding qualitative data (22). The analysis team met regularly to discuss new codes or emerging questions and shared interim findings with the Steering Committee to guide interpretation. Coding was conducted by three researchers in Atlas.ti (23). Study reporting is consistent with the Standards for Reporting Qualitative Research (SRQR) checklist (24).

## Results

### Participants

We interviewed 50 adults diagnosed with confirmed high cholesterol at least 2 years prior (mean age: 54.7 ± 10.4 years; 50% women). Although the original recruitment target was 45 participants, we enrolled an additional five individuals to account for logistical challenges related to screening and scheduling interviews across multiple international time zones. Our final sample includes 15 people from Brazil, 16 people from the United States, and 19 people from Australia. Of these, 22 (44%) experienced an ASCVD event at least 1 year prior and 1 year following diagnosis (Table 1). The majority had never smoked (*n* = 33), though half were overweight (*n* = 24), and many reported comorbid hypertension (*n* = 33) or diabetes (*n* = 20). More than half of the participants were currently married (*n* = 27), had at least a college degree (*n* = 31), and lived in a suburban community (*n* = 32). A high-level summary of thematic findings is presented in Table 2.

**Table 1:** Characteristics of study participants.


VARIABLE	TOTAL		AUSTRALIA		BRAZIL		UNITED STATES	

n (%)	50		19	38%	15	30%	16	32%

ASCVD (n, %)								

Yes	22	44%	7	37%	7	47%	8	50%

No	28	56%	12	63%	8	53%	8	50%

Age (mean, SD)	54.7	10.7	61.9	11.5	48.3	11.5	51.2	10.1

Age Category (n, %)		0%						

Less than 45	12	24%	2	11%	7	47%	3	19%

45 to 64	27	54%	8	42%	8	53%	11	69%

Over 65	11	22%	9	47%	0	0%	2	13%

Sex (n, %)		0%		0%		0%		

Female	25	50%	4	21%	10	67%	11	69%

Male	25	50%	15	79%	5	33%	5	31%

Marital Status (n, %)								

Married	27	54%	9	47%	11	73%	7	44%

Never Married	14	28%	8	42%	2	13%	4	25%

Divorced	7	14%	1	5%	2	13%	4	25%

Widowed	2	4%	1	5%	0	0%	1	6%

Smoking (n, %)								

Never smoked	33	66%	10	53%	9	60%	14	88%

Current smoker	7	14%	3	16%	3	20%	1	6%

Ever smoker	10	20%	6	32%	3	20%	1	6%

Weight (n, %)								

Underweight	0	0%	0	0%	0	0%	0	0%

Normal weight	17	34%	6	32%	7	47%	4	25%

Overweight	24	48%	13	68%	5	33%	6	38%

Obese	9	18%	0	0%	3	20%	6	38%

Rurality (n, %)								

Rural	3	6%	1	5%	0	0%	2	13%

Suburban	32	64%	18	95%	5	33%	9	56%

Urban	15	30%	0	0%	10	67%	5	31%

Education (n, %)								

Some high school	4	8%	4	21%	0	0%	0	0%

High school	5	10%	1	5%	4	27%	0	0%

Some college	10	20%	1	5%	2	13%	7	44%

College graduate or above	31	62%	13	68%	9	60%	9	56%

Event (n, %)								

Ischemic stroke	5	10%	0	0%	2	13%	3	19%

Heart attack	14	28%	4	21%	5	33%	5	31%

Peripheral artery disease	7	14%	3	16%	2	13%	2	13%

Unstable Angina	3	6%	0	0%	2	13%	1	6%

Comorbidity (n, %)								

Diabetes	20	40%	4	21%	10	67%	6	38%

High blood pressure	33	66%	10	53%	12	80%	11	69%


**Table 2:** Summary of study findings.


TOPIC	KEY TAKEAWAYS

Pathways to diagnosis	In most instances high LDL-C was diagnosed as part of a routine health check-up; however, one-quarter of participants did not access health system services until they experienced signs and symptoms associated with ASCVD or a symptomatic co-occurring condition such as diabetes. The remaining participants were diagnosed during routine care for a comorbid condition, most frequently diabetes.

Understanding of LDL-C risks and treatment	One in three participants described not fully understanding how serious high cholesterol was after being diagnosed. Only around half of participants were aware at diagnosis that lipid-lowering therapies need to be taken throughout their life. Among people who experienced an ASCVD event, the majority did not immediately recognize their symptoms as heart-related, sometimes waiting hours or days before seeking care.

Emotional impacts	Learning of a high LDL-C level often triggered emotional responses. Many patients described feeling anxiety or fear about developing heart disease, especially if they understood the link between cholesterol and serious events. Some who had struggled with high cholesterol for years (or had family histories of heart disease) expressed frustration or fatalism, feeling that a heart problem might be inevitable. These emotions influenced how patients approached their treatment.

Factors impacting lifestyle change	Nearly all patients reported attempting lifestyle modifications (such as healthier diets, weight loss, and exercise) to improve their cholesterol. However, they encountered significant challenges in sustaining these changes. Common barriers included conflicts between work schedules and lifestyle change, difficulty altering long-standing eating habits, limited knowledge about heart-healthy diets, cultural or regional dietary preferences that are hard to change, lack of time for exercise, and in some cases, disappointment when lifestyle changes alone did not quickly lower LDL-C.

Perceptions of current therapies and desired outcomes	Participants expressed overall satisfaction with current treatments but envisioned ideal therapies like those requiring less frequent administration, offering non-pill formats (e.g., injections and patches), and having fewer side effects, better efficacy, or greater affordability. Meaningful treatment outcomes extended beyond clinical metrics. Patients valued improvements in lab results, prevention of cardiovascular events, longevity, and enhanced quality of life, particularly in maintaining independence and spending time with loved ones.


### Pathways to the diagnosis of high cholesterol

Participants described three main pathways to becoming diagnosed with high cholesterol. Routine physical exams conducted by primary care providers were the most common diagnostic method, accounting for half of all diagnoses. However, approximately one-fourth of diagnoses were prompted by symptomatic presentations or incidental findings during emergency visits. The third path to diagnosis was through a healthcare visit for another condition, frequently diabetes. People in all three countries described all three pathways to diagnosis (Supplementary Table 1). Across all countries, diagnoses were made through lab tests, though the timing of these tests varied, occurring before, during, or after the diagnostic visit. Initial discussions about lab results and treatment options typically took place during the same visit, follow-ups, or via online portals.

Over half (*n* = 27) of participants were prescribed statins immediately, while others were advised first to attempt lifestyle changes. In cases of persistent high cholesterol levels, statins were later initiated for an additional eight patients. Participants described a range of emotional responses to their diagnosis. Many reported feeling overwhelmed (*n* = 13, 26%) or surprised (*n* = 7, 14%), particularly when managing other diagnoses or when they were unfamiliar with the medical terminology used by healthcare providers (see Table 3). Concerns about their risk for cardiac events added to their emotional burden.

**Table 3:** Example messages to improve messaging on the importance of remaining adherent to a care plan suggested by participants.


Message Recommended by Participants	Illustrative Quotes from Interviewees

Share examples from real people living with high cholesterol	*Participant in the United States who experienced an ASCVD event‬ hospitalization:* I would start off with the education part of lowering your cholesterol could significantly lower your chances for heart disease, heart attack. I would try to give examples of things that I have done myself and show little snippets of—or videos of me actually doing these things. And I guess trying to appeal to the sense that I’m living. Had I not been taking these medications, or if I’m not taking it correctly…what the effect of that would be.‬‬‬

Preventing a cardiac event is easier than recovering from one	*Participant in the United States who experienced an ASCVD event‬ hospitalization:* Don’t wait until you’ve fallen off the cliff to try to get back up. So be careful. Be careful, because then it’s much harder to go back and try to fix it than it is to prevent it.

Take it seriously because the effects of not taking it seriously are physically painful	*Participant in Brazil who experienced an ASCVD event hospitalization:* Look, I know I felt terrible. It felt like death. So whoever can, whoever has a health plan, whatever, get yourself treated, go and get your tests done to make sure you don’t have anything, to take care of yourself because it’s very bad. It’s terrible.

Be blunt about risk of cardiac events	*Participant in Australia who had not experienced an ASCVD event hospitalization:* I think, personally, the world in general has become too afraid to be direct, do you know what I mean? I think the message doctors need to give is direct to the point and even throw a little bit of scare factor in. And come to think of it, [doctor’s name] did say it could lead on to heart disease and he did also mention about my weight. He always mentions my weight and he’s given me charts of what I should eat and what I shouldn’t. I think people just need to realize, look, if you’ve got it, take your tablets. It’s a very simple regime to do and it becomes automatic and hopefully doesn’t lead to… I don’t know. You basically really gotta be more blunt and say, Okay, if you don’t take your tablets, you’re gonna have the heart attack and you’re gonna end up with problems with your heart. So, I don’t know. It’s hard. It’s very hard to say.

You need to take your medicines	*Participant in the United States who experienced an ASCVD event‬ hospitalization:* You just have to try and live every day the best that you possibly can. And I know that, for a lot of people, they don’t like taking meds. But they are a life-saving tool. There’s no question about it.

Repetition	*Participant in the United States who experienced an ASCVD event‬ hospitalization:* Probably in my case, it’s effective just to hear it all the time. I can be stubborn and tend to do things at my own pace. So, I’m the kind of individual that constantly needs to hear it, probably, for it to be most effective.


Some participants, especially those with family histories of unmanaged cholesterol, recognized the seriousness of their diagnosis early on. For instance, one Australian participant attributed his proactive approach to witnessing his mother’s long-term paralysis following a stroke:

So, I knew that from my mother’s case, right? She had high cholesterol and then she got stroke, so I knew that was the evidence that I had to be very careful and very alertful about the potential implication on my health.

—Participant from Australia who has not experienced an ASCVD event.

For others, the perceived severity of their condition was influenced by their healthcare provider’s communication style. Direct and clear discussions linking high cholesterol to cardiac events were effective for some, while others misinterpreted lifestyle modification recommendations as an indication that their condition was not serious. In certain cases, participants attributed their diagnosis to temporary factors, such as recent dietary habits, or dismissed it as an expected consequence of aging. A subset of participants (*n* = 5) expressed embarrassment, linking their diagnosis to neglect of their health prior to diagnosis.

### Pathways to diagnosis and management of ASCVD events

Among participants who experienced an ASCVD event at least a year before the interview and at least 1 year after their diagnosis with high-LCL-C (*n* = 22), initial symptoms included flu-like sensations, chest pain, fatigue, nausea, and paleness. However, 68% did not recognize these symptoms as heart-related and often delayed seeking care. Some participants waited hours or days, assuming their symptoms were less serious. For instance, one participant in Brazil described experiencing symptoms for ten days before finally driving to the emergency room after friends commented on his appearance:

I had high cholesterol for years and years and years. It hasn’t changed, regardless of taking medication, watching my diet or anything, just never changed, never went down. And it slowly crept up a little bit and before I knew it, I was having a quadruple bypass.

—Participant from Australia who experienced an ASCVD event.

In some cases, family members or colleagues played a crucial role in helping participants enter the healthcare system (*n* = 4) after noticing symptoms. These people often convinced participants to seek care or called emergency services on behalf of participants. Participants who sought care promptly described either directly going to the emergency department or being referred after visiting primary or urgent care.

### Emotional and cognitive reactions to ASCVD events

Participants frequently described feeling surprised and scared during their ASCVD events, with many fearing death and worrying about their families’ futures. Some described delaying care because they did not want to disrupt work, family life, or other activities, reflecting a tension between personal health and external responsibilities. Following hospitalization, participants often had difficulty recalling specific details of their care due to medication effects, the emotional impact of the event, unconsciousness, or the passage of time.

### Follow-up care after ASCVD events

Post-event care often involved referrals to primary care providers or cardiologists, with the latter playing a more prominent role in participants’ care than before the event. Some participants reported changes in their care team, with about one-third changing physicians (*n* = 8) and half adding a cardiologist (*n* = 11). The speed of arranging follow-ups varied, ranging from a few days to over a week.

During follow-up appointments, participants described discussions about medications and lifestyle changes. These appointments often marked a turning point, as participants reflected on the need to prioritize their health. Despite this, challenges such as maintaining lifestyle changes, coping with inconsistent lab results, and adhering to complex treatment plans persisted.

### Burden of managing high cholesterol: Awareness, lifestyle changes and challenges

#### Awareness of LDL-C targets

Most participants (*n* = 30) were unfamiliar with their specific LDL-C target values, both at diagnosis and throughout their experiences managing high cholesterol. Instead, they relied on alternative methods to interpret lab results, such as color-coded indicators provided by their healthcare portals or guidelines found online. Many participants depended on their healthcare providers (HCPs) to explain the significance of their cholesterol levels. One participant shared:

When I go to my little portal, all the results are there. And I can look at it and if it’s too high, it’s in red. If it’s at a good level, it’s black. If it’s too low, it’s, I think, yellow. […] But I don’t have those in my mind. Just like blood pressure, I have no clue what a normal blood pressure is because I have high blood cholesterol.

—Participant from the United States who experienced an ASCVD event.

#### Awareness and understanding of lifelong therapy

Before experiencing an ASCVD event, nearly half of the participants (*n* = 23) were unaware that lipid-lowering therapies would need to be taken throughout their lives. Among those who were aware, knowledge often stemmed from HCP discussions at diagnosis, personal experiences (e.g., a family member’s health), or professional exposure to medical information. Some participants expressed frustration over their misunderstanding, as they believed that weight loss or other lifestyle changes would allow them to discontinue their medications:

I was under the impression that we’re going to do some more blood work in a year or so and that you can be off of it… But that never happened.

—Participant from the United States who experienced an ASCVD event.

#### Medication adherence strategies

Participants employed various strategies to ensure adherence to their prescribed medications. Common techniques including using weekly pill organizers, adhering to a daily routine, and designating specific medication storage locations. These strategies were generally effective unless participants faced disruptions in their routines due to travel or work:

I package [my pills] together in these little pill packs and I do it once a month. In the morning, I just grab that day’s little box and take my morning meds… But sometimes I get caught up and busy and I notice the box is still sitting there come lunchtime.

—Participant from the United States who has not experienced an ASCVD event.

#### Changes in medication use after an ASCVD event

Following an ASCVD event, participants described changes in their medication regimens and attitudes toward adherence. Some initiated statin therapy for the first time (*n* = 8), while others remained on their existing medications without dose adjustments (*n* = 5), switched medication classes (*n* = 4), or increased dosages (*n* = 2). Statins, either alone or in combination, remained the cornerstone of therapy for most participants post-event (statin alone, *n* = 15; statin in combination, *n* = 2). A small number of participants (*n* = 2) reported being prescribed PCSK9 inhibitors, although one discontinued use due to side effects.

Participants also reported starting additional medications to manage comorbid conditions, such as ACE inhibitors for hypertension or anticoagulants to prevent future strokes. These changes often reflected a shift in care priorities after a cardiac event, with participants taking a more active role in managing their health:

From that moment on [the heart attack], I became a saint. I took the right medication, I did everything right, you know? […] I changed completely. At that time, it was physical exercise, walking, eating lighter.

—Participant from Brazil describing post-event changes.

#### Lifestyle changes and challenges

Approximately two-thirds of participants (*n* = 33) reported attempting lifestyle changes after their high cholesterol diagnosis, including eating more healthily, reducing alcohol consumption, quitting smoking, losing weight, or reducing stress. However, adherence to these changes was often influenced by personal and environmental factors. For example, a participant in Brazil described losing weight while continuing to smoke and drink, ultimately experiencing a heart attack.

Participants frequently emphasized the role of family dynamics in adopting and sustaining dietary changes. While family support facilitated success for some, others encountered resistance. One participant described the difficulty of coordinating separate healthy meals for themselves when other family members preferred less healthy foods. Similarly, parents faced challenges preparing meals that balanced their dietary needs with their children’s preferences.

Work schedules and daily routines also posed significant barriers to maintaining healthy habits. Busy schedules often led participants to rely on dining out, where they had limited control over food preparation. One participant explained:

Because of our lifestyle at the time, I was traveling for business 150,000 miles a year, a lot. […] We were eating out almost every day and when you eat out, you can’t control what they’re putting in the food. […] Even if you get a salad, it’s covered in some dressing that’s 50% oil.

—Participant from the United States who experienced an ASCVD event.

Despite these challenges, participants described several strategies to implement and sustain lifestyle changes. These included building time for self-care into their routines, such as setting aside time for exercise or meal preparation and seeking guidance from healthcare professionals like physical therapists and nutritionists. For instance, one participant reported initial shortness of breath and fatigue when beginning an exercise regimen but worked with a physical therapist to gradually increase their activity to one hour, five days a week.

#### Emotional responses and long-term challenges

While many participants initially adhered diligently to medications and lifestyle changes post-event, some described difficulty maintaining these behaviors over time, especially when lab values did not improve despite their efforts. This lack of progress led to feelings of frustration and discouragement:

I was told at one point to lose at least 40 pounds. I did that two years ago… And yet my numbers were still elevated. So, I don’t personally see it like that was a success, because it didn’t really knock my numbers down at all.

—Participant from the United States who experienced an ASCVD event.

Participants in Brazil frequently noted that their HCPs emphasized lifestyle modifications over adjusting medication regimens, which some found disheartening:

I have never stopped taking my medication. I’ve never gone a day without taking my medication. […] But my cholesterol numbers didn’t change.

—Participant from Brazil who experienced an ASCVD event.

Motivational barriers were also common, particularly during the initial phases of implementing lifestyle changes. Participants described physical discomfort, such as shortness of breath during exercise, or psychological barriers like self-criticism and a lack of discipline:

Part of it is laziness. The other part is because I haven’t been active in so long, that when I do start to walk or do those types of things, I get out of breath quickly and that discourages me from continuing.

—Participant from the United States who has not experienced an ASCVD event.

### Patient-focused drug development

#### Perspectives on treatments

When asked about a perfect treatment that was not a cure, sixteen participants described being satisfied with their current treatment. The most frequent suggestion for improving current therapy was reduced administration frequency (*n* = 21), with responses ranging from weekly to annually. Non-tablet forms were the second-most requested attribute of an ideal treatment (*n* = 16). Injections were the most frequently described specific form (*n* = 6), but other non-tablet or pill forms (e.g., liquid, patch, implantable) were mentioned by ten people. People also described their ideal treatment as having reduced side effects relative to their current therapy (*n* = 11); additional features of an ideal treatment included improved efficacy over current therapies (*n* = 10), combination therapies for managing comorbid conditions (*n* = 6), and affordability (Supplementary Table 2).

#### Patient-centered outcomes and meaningful treatment benefits

Participants identified a range of meaningful outcomes that motivated them to adhere to their treatments and lifestyle changes, highlighting both clinical and personal priorities.

- **Improvements in lab values:** Participants frequently emphasized the importance of seeing tangible improvements in their lab results. These served as a validation of their efforts and a sign that they were reducing their risk of adverse outcomes.
- **Avoidance of ASCVD events:** Preventing an ASCVD event that required hospitalization was a top priority, regardless of whether participants were seeking to avoid an initial or subsequent event. Participants associated successful treatment with the ability to maintain their cardiovascular health over the long term.
- **Long life:** Many participants expressed a desire for longevity, often framing this goal in terms of spending more time with loved ones, including family and friends. Longevity was linked to a broader desire for an active, engaged life.
- **Improved quality of life:** Participants also highlighted the quality of life as a critical outcome, describing goals such as engaging in daily activities outside the home, traveling, and increasing physical activity and energy levels.

#### Patient recommendations on communicating about LDL-C and ASCVD

Participants shared strategies to improve communication about high LDL-C and ASCVD risk. They emphasized authenticity, relevance to individual circumstances, and clear language. One recommendation was to include real-life stories from people with high LDL-C, especially those who had experienced a cardiovascular event. These stories could help others understand the risks of not treating high LDL-C and the benefits of staying on medication.

Several participants, especially those who had been hospitalized for an ASCVD event, supported using direct, emotionally intense messages. They pointed to the value of honest descriptions of the pain and fear linked to heart events to encourage action. Some said blunt language about risk, what one called a ‘scare factor’, can be effective when combined with clear steps for prevention, such as viewing medication as a life-saving tool.

Participants also noted that repetition matters. Hearing the same messages across different settings helped reinforce the importance of treatment, particularly for those who tend to put it off.

## Discussion

This pilot study, led by a patient organization and guided by a multi-stakeholder steering committee, provides valuable insights into the diagnostic pathways, patient experiences, and management challenges associated with high cholesterol and ASCVD across diverse healthcare systems in Australia, Brazil, and the United States. The findings highlight the importance of incorporating patient perspectives on healthcare experiences, treatment barriers, and communication gaps. They can help guide the co-development of clinical decision support tools, provider education, and patient communication strategies that may improve the effectiveness of both medication-based and non-medication interventions.

While routine physical examinations by primary care physicians were the predominant method for diagnosing high cholesterol, one-fourth of diagnoses resulted from symptomatic presentations or incidental findings during emergency visits, with routine screenings being less common. These experiences highlight opportunities for healthcare policymakers to prioritize preventive health services when designing cardiovascular health programs. For example, Brazil recently initiated an effort to establish a national cardiovascular plan, and the European Union announced that a new European Cardiovascular Health Plan will be developed (25).

Across all countries, participants reported a range of emotional responses upon diagnosis, including feelings of being overwhelmed, particularly when managing multiple health conditions. How healthcare providers communicated the diagnosis significantly shaped patients’ perceptions of the seriousness of their condition. Clear, empathetic, and tailored communication is essential in fostering a proactive approach to managing high cholesterol (27). Integrating plain-language communication strategies into clinical practice guidelines and shared decision-making tools is crucial to ensuring consistent, patient-centered messaging (28).

Many participants lacked detailed knowledge of their specific target values and often relied on lab results for interpretation. This gap in understanding is a reminder that lab reports and conversations with people with high cholesterol must effectively convey personalized target values and contextualize these within the patient’s broader cardiovascular risk profile, rather than relying on population averages. This is particularly complex in the context of accurately communicating LDL-C target values, since various factors, including genetics, impact targets (7). Incorporating visual aids and patient-friendly language into lab reports could bridge this knowledge gap and empower patients to engage in their care actively.

Moreover, while two-thirds of participants attempted lifestyle modifications, sustaining these changes was challenging due to barriers such as family dynamics, work schedules, and competing priorities. These challenges align with previous studies highlighting obstacles to lifestyle modification in chronic disease management. For instance, a recent systematic review identified barriers, including lack of knowledge and training, staffing shortages, insufficient reimbursement for lifestyle modification programs, and skepticism regarding the effectiveness of non-medical interventions (29). Addressing these barriers requires a multipronged approach, including adopting a patient-centered approach that results in a co-created care plan. Increasing provider training on behavioral counseling, enhancing the availability of structured lifestyle intervention programs, and advocating for policy changes to improve reimbursement for non-pharmacologic interventions can help to support the development of these plans.

Finally, our findings suggest that a multidisciplinary approach involving dietitians, exercise physiologists, and psychologists may be particularly effective in supporting sustained lifestyle changes. This aligns with a need for patient-centered, co-developed solutions guided by patient input (26).

## Future Research

Future studies should identify which treatment attributes and outcomes patients value most and examine how experiences differ by gender, community setting, and length of time on therapy. Researchers should also test whether using patients’ recommended approaches for explaining LDL-C risks improves understanding, adherence, and ultimately health outcomes.

## Strengths and Limitations

This research has several key strengths, most notably, it was patient-led and utilized a patient-centered approach. The research team interviewed 50 people in three countries with confirmed diagnoses of high cholesterol. The Global Heart Hub, a patient organization, initiated and led the research. All study procedures, from conceptualization to dissemination, were guided by a Steering Committee that included a patient, patient advocates for the specific countries of the study, cardiologists, and researchers. The interview discussion guide was developed using the NHC’s PEMT, the output of a collaboration between the patient community and researchers. All interviews were conducted in participants’ native language by a native speaker to ensure findings accurately reflect patients’ experiences and priorities.

Despite these strengths, the study findings must be considered in light of limitations. Participants described their past experiences, which may be subject to recall bias. Participants’ experiences may not be generalizable to all people with elevated LDL-C or ASCVD. For example, the eligibility criteria did not allow participants who received their first elevated LDL-C diagnosis only after experiencing an ASCVD event. The study also recruited participants primarily from urban and suburban settings, and the experiences of these people may not align with the reality of living with high cholesterol for people living in rural areas.

## Conclusion

Patients have a contribution to make by sharing their experiences, which cannot come from anyone else. This study provides insights into patients’ experiences living with and managing elevated LDL-C and ASCVD. It provides insights into potential opportunities for policymakers and healthcare providers to improve the detection of elevated LDL-C. It can also inform future interventions to support patients in understanding risks and strategies for reducing the risk of ASCVD events.

## Additional Files

The additional files for this article can be found as follows:

10.5334/gh.1441.s1 Supplementary Appendix A. Steering Committee Members.

10.5334/gh.1441.s2 Supplementary Appendix B. Full interview Guide.

10.5334/gh.1441.s3 Supplementary Table 1. Participant-described pathways to diagnosis.

10.5334/gh.1441.s4 Supplementary Table 2. Participant-described attributes of an ideal treatment based on openended questions.
