# Supplementary material for: Experiences of People Diagnosed with High Levels of LDL Cholesterol and Atherosclerotic Cardiovascular Disease: Results from a Multinational Qualitative Study
Source: Glob Heart. 2025 Jul 15;20(1):63. doi: 10.5334/gh.1441 (PMC12273682; doi:10.5334/gh.1441)
Supplement: Supplementary Appendix A. — Steering Committee Members. [file gh-20-1-1441-s1.pdf]

Supplementary Appendix

Supplementary Appendix A. Steering Committee Members

Fernanda de Carvalho
Instituto Lado a Lado Pela Vida (Brazil)

Celina Gorre
WomenHeart (United States)

Tanya Hall
hearts4heart (Australia)

Susan Hennessy, PhD, MHSc, BSc(Hons)
University of California, San Francisco

Dhruv S. Kazi, MD, MSc, MS
Harvard Medical School

Kornelia Kotseva, MD, PhD, FESC
National University of Ireland – Galway

Patsy Petrie
HEART UK & FH Europe Foundation Ambassador
