# Supplementary material for: Experiences of People Diagnosed with High Levels of LDL Cholesterol and Atherosclerotic Cardiovascular Disease: Results from a Multinational Qualitative Study
Source: Glob Heart. 2025 Jul 15;20(1):63. doi: 10.5334/gh.1441 (PMC12273682; doi:10.5334/gh.1441)
Supplement: Supplementary Appendix B. — Full interview Guide. [file gh-20-1-1441-s2.pdf]

Supplementary Appendix B. Full interview Guide

Pre-event experience with high LDL-C

**Before I ask you about your experience with [cardiac event/condition], I would like to know more about your experience with high cholesterol before you had [cardiac event/condition]**	
**LDL-C: diagnosis**	Could you please tell me about when you first found out you have high cholesterol? How long ago was that?<br>• Did you find out that you had high cholesterol during a regular check-up or were you at the doctor's office for another reason?
**LDL-C: before event/ shared decision-making**	Could you describe conversation(s) between you and your doctor after finding out you had high cholesterol, but before you had [cardiac event/condition]. For example, did you discuss risks of high cholesterol?<br>• Did you discuss how to manage high cholesterol? What did you discuss?<br>• Did you take the link seriously? Why or why not?
**LDL-C: medicine before event**	Did you start taking medicine? Why or why not?<br>• Do you recall what medicines you started taking at that time?<br>• Were you aware that medicines for high cholesterol need to be taken throughout your life?<br>Note to reviewers: there is a more detailed section on treatments in the "living with" section.
**LDL-C: medicine before event (adherence)**	Thinking back, did you take your cholesterol medicine according to your doctor's instructions?<br>• Did you doctor discuss potential health consequences of not staying on treatment?<br>• Did you ever miss doses of your medicine? Could you tell me more why the medicine(s) was not easy to take?
**Lab testing before event**	How often did you get lab tests for cholesterol?<br>• Did you know what your cholesterol levels should be?<br>• Who reviewed the results with you? Did the results make sense to you? Why or why not?
**Lifestyle changes**	Did you make any lifestyle changes to help you manage high cholesterol? What changes did you make?<br>Examples include:<br>o A healthy diet that is low in salt<br>o A healthy diet that is low in fat/saturated fat<br>o A healthy diet that includes more fish<br>o A healthy diet that includes less sugar<br>o A healthy diet that includes more fruits and vegetables<br>o Exercise<br>o Maintaining a healthy weight<br>o Stopping smoking<br>o Reducing or trying to manage stress<br>What made you decide to make those changes? Who helped you decide on those lifestyle changes?

Comorbidities

**Other diagnoses**	Still thinking about your experience before having a [cardiac event/condition], did you have any other health conditions besides high cholesterol? Which health conditions?<br>• How long have you had [other dx]?

Life Factors

**Point to "life factors" on the map.**	
**Life factors impact on seeking care**	• Did the life factors impact your experiences with managing high cholesterol before [cardiac event/condition]?

Closing Pre-Event High Cholesterol

	Do you have anything else you'd like to tell me about your experiences with high cholesterol before your [cardiac event/condition]?

Seeking care for cardiac event/condition

**Decision to seek care**	Thank you very much for answering my questions. I would now like to ask you more about your experience with [cardiac event/condition]. To start off, I was wondering, what was the thing or thing(s) that made you go to the doctor?<br>○ Did something feel different or not quite right, or did you see a healthcare provider for another reason?<br>▪ What did you notice? What did it feel like?<br>▪ How long did the [symptoms] last? How often did they occur?
**Provider type – decision to seek care for cardiac event**	Once you decided to seek care, could you tell me about where you went first (e.g., clinic, hospital, etc.)?

Ask about their experiences getting diagnosed with cardiac event/condition

**Seeking care**	What happened during the appointment/emergency/hospital admission?
**Lab tests and high cholesterol-cardiac event**	Did you have any lab tests? If so, when did you receive the results?<br>• Did the results make sense to you?<br>• Do you recall if you had cholesterol tests done at the hospital?
**Follow-up care after cardiac event**	What instructions did you have for follow-up care? For example, did the [provider type] recommend that you go to see somebody else next, such as a specialist?<br>• Did you decide to go? Why or why not?<br>• How long did it take after your first visit with the [provider type] to see this [new provider type]?<br>• Did any of the life factors impact your decision to follow-up?
**Questions after cardiac event**	Did you feel like you left the office/emergency department/hospital with an understanding of [Cardiac event/condition]?<br>• What questions did you have?<br>• Where did you look to find information following your appointment?

Emotional Health & Awareness of Link Between High Cholesterol and Heart Disease

**Emotional health after cardiac event**	How were you feeling emotionally after having the [cardiac event/condition]? Some people have mentioned feeling calm, worried, overwhelmed, relieved, or angry.<br>• Did you feel motivated to treat and monitor your cholesterol levels? Why or why not?
**Awareness of heart disease before event**	What did you know about your heart disease risk before your [cardiac event/condition]?<br>• Did your doctor explain your overall heart disease risk to you before your [cardiac event/condition]? How?<br>• Do you have a family history of premature cardiac events/conditions? [men under 55 and women less than 65]<br>• What do you know now that you wish you had known then?

Closing before living with a diagnosis

	Before we move on, do you have anything else you would like to tell me about what it was like getting diagnosed with [cardiac event/condition/ event]? High cholesterol?

Ask about their experiences living with cardiovascular disease / high cholesterol

**Point to the green icon "living with a diagnosis" on the map.**	
**General experiences since cardiac event**	Could you please tell me more about your experiences since you first got a diagnosis with [cardiac event/condition]?
**Health care routine**	Which health care providers do you typically see since you received a diagnosis? Tell me about your usual routine.<br>• What kind of specialists do you see? How often do you see them?<br>○ What do you discuss during appointments with your specialists?<br>• Do you see healthcare providers for reasons besides high cholesterol and [cardiac event/condition]? Please describe which other providers you see.

Treatment they are currently getting.

**Point to "Tried a treatment" on the map.**	
**Post event Treatment initiation: Shared decision-making**	• Could you please describe what treatment(s) – if any - are you currently taking/getting for your high cholesterol?<br>• What medicines have you taken in the past?<br>• When you were prescribed the medication, what was the conversation like between you and your doctor?<br>○ Did he/she talk about other medications? Or give you options?<br>• Are the discussions you have with your doctor about managing high cholesterol different now than they were before you had the [cardiac event/condition]? How so?

**Cholesterol Goals & Lab testing**	Do you know your cholesterol goal or target numbers? Who told you about them?<br>• How often do you get lab tests for cholesterol? Can you tell me more about that process?<br>• Who reviews the results with you? Do the results make sense to you? Why or why not?<br>• How confident are you that you can control your LDL cholesterol level to target by taking medications?
**Treatment benefits**	• What are the biggest benefits of the treatment?
**Treatment downsides**	• What are the biggest downsides or not-so-good things about the treatment?<br>• Do you have any side effects from treatment(s)?
**Treatment alternatives**	• What other treatments for high cholesterol do you know about?<br>• How did you learn about the different treatments available to you?<br>• Have you discussed alternatives with your health care provider?
**Interactions with other medicines**	• Has the medicine you are currently taking, or one you took in the past, interacted with other treatments you're taking for something else? For example, have there been any unusual side effects that could be related to taking 2 different medicines together?

Adherence to medicine

**Adherence to medicine**	• Do you ever miss doses of your current medicine? If you do, on average how many doses of your medicine would you say you miss in a week?<br>o Can you tell me more why the medicine(s) is/are not easy to take?<br>• How about past medicines?
**Strategies for improving adherence to medicine**	• What do you think would make it easier for you to take your current or past medicines as prescribed?<br>• Have you tried any strategies to help you take your medicines according to your doctor's instructions? What have you tried?<br>o Examples: day-of-the-week pillboxes, apps on your cell phone or an alarm, taking medicine at the same time every day<br>• How did [strategy] help you?<br>• How long did you stick with that strategy?
**Making permanent changes**	• Are there habits you think you could adopt to take your medicine as prescribed every day? What are they?
**Perspective towards medicine following cardiac event**	Have your feelings towards managing high cholesterol changed since the [cardiac event/condition]? How so?<br>• What do you do differently now than before?<br>• Are there things you wish you had known before the cardiac event that might have made you more likely to take your medicines or stick to lifestyle changes? What are they?

Risk factors for heart disease

**Second probe on connection between heart disease and high cholesterol**[1]	• Do you know if you have any risk factors for heart disease? What are they?<br>• Have you ever had any discussions with your doctor about heart disease risk or risk factors?<br>○ If yes - tell me about what your doctor said?<br>○ If no - have you ever had any discussions about blood pressure, cholesterol, smoking, diet or exercise?

Lifestyle changes

**Lifestyle changes**	• Have you made any lifestyle changes to help you manage high cholesterol? What changes have you made?<br>Examples include:<br>○ A healthy diet that is low in salt<br>○ A healthy diet that is low in fat/saturated fat<br>○ A healthy diet that includes more fish<br>○ A healthy diet that includes less sugar<br>○ A healthy diet that includes more fruits and vegetables<br>○ Exercise<br>○ Maintaining a healthy weight<br>○ Stopping smoking<br>○ Reducing or trying to manage stress<br>• What made you decide to make those changes? Who helped you decide on those lifestyle changes?
**Adherence to lifestyle changes**	• Have you been able to stick with [lifestyle change]? Why or why not?<br>○ Have you tried any strategies to make it easier to stick with [lifestyle changes]? What have you tried?<br>• What would make it easier for you to stay on track?<br>○ What do you feel would encourage you or others to take a proactive approach to cardiovascular health?
**Motivators to stick with lifestyle changes**	• What would make it easier for you to permanently adopt [lifestyle changes]?<br>○ Have you noticed any changes from sticking with lifestyle changes? For example, changes in your lab results?

**Trustworthy messengers**	Are there particular people or institutions that you are most likely to listen to when it comes to adopting lifestyle changes or taking your medicines every day? Who are they?
**When will messages resonate**	At what points across your health journey, for example when you first got diagnosed with [cardiac event/condition] or high cholesterol, or at another point, do you think it would be most effective to hear from them?
**What messages?**	If you were developing a public service or educational campaign to encourage people with high cholesterol to make lifestyle changes or take their medicines as directed, what messages would you use?

Ideal treatment

[1]Several questions on risk of heart disease are adapted from the "Healthy Heart Study: Improving doctor and patient decision making about cardiovascular disease (CVD) risk assessment and monitoring."

**Ideal treatment**	• If you had a perfect treatment that wasn't a cure, what would it do for you? How would your life improve if you had a more effective (or better) treatment?<br>• If you could wave a magic wand and change something about the medicine you are currently taking, what would it be?<br>○ For example, how easy it is to take the medicine or how the medicine makes you feel.

Wellbeing and burden of disease

**Well-being & high cholesterol**	Please think about the daily activities that are important to you or that you like to do. How has [cardiac event/condition] or high cholesterol impacted your ability to those things?<br>• Has your diagnosis with high cholesterol affected your family or other loved ones?<br>• Has your diagnosis with high cholesterol affected your quality of life?<br>• How has your [cardiac event/condition] affected your family or loved ones?
**Worry**	What worries you most about your high cholesterol? How about previous [cardiac event/condition]?

Care Coordination & Multiple Chronic Conditions

**Coordination of care**	Do you have other chronic conditions besides [cardiac event/condition]? How do you coordinate your care for these conditions?<br>• How has having multiple chronic conditions affected your life?<br>• Do your providers communicate with each other and coordinate your care when exploring treatment options?<br>• Has having multiple chronic conditions impacted your treatment decisions?<br>• Have you had any negative effects occur because your care was not coordinated?<br>• Please describe for me how having more than one high cholesterol affected your<br>○ finances<br>○ profession or career<br>○ family<br>○ lifestyle<br>○ mental or emotional well-being

Satisfaction with care & quality

**Satisfaction with care**	How satisfied are you with the health care you receive? What makes you say that? Your comments are kept private.<br>• Do you believe your providers understand what is important to you – like things you want to see happen in your life? Feel free to talk about past and current providers.
**Quality**	Have you had any positive or negative experiences you would like health care providers and researchers to know about? This could be anything to do with your high cholesterol such as an experience with symptoms, medication, health care providers, stigma or bias, etc.<br>• When you think about the health care system, what do you think are signs of “good” quality care?<br>• And what do you think are signs of “bad” quality care?

Desired Outcomes and Life Aspirations

**Outcomes**	Which outcomes are most important to you when it comes to your high cholesterol or heart health?<br>• Outcomes might relate to how you feel, for example, fewer symptoms; how you function, such as being able to exercise or walk to work; or living longer.
**Aspirations**	How have your aspirations changed because of [cardiac event/condition] or high cholesterol? How have they changed across the map over time?

Questions & Information sources

**Outstanding questions**	• What questions do you still have about [cardiac event/condition] or high cholesterol?
**Information sources & resources**	• Where do you look for information about [cardiac event/condition] or high cholesterol?<br>• What information did you find? Did the information that you found answer your questions?<br>• What sources of information did you trust or rely on most?<br>• Are there resources you wish existed, but you haven’t been able to find?

Life Factors

**Point to “life factors” on the map.**	
**Life factors (living with)**	How do the “life factors” affect your experience with [cardiac event/condition] or high cholesterol?<br>For example: lack of insurance, cost concerns, not being able to take off work

Closing

**Final thoughts**	Is there anything else you would like to add about your experience with heart health or high cholesterol that we didn't touch on, but you think is important?
**Recommendations to other stakeholders**	Is there anything else you would like to tell **researchers** about having [cardiac event/condition] or high cholesterol?<br><br>Is there anything else you would like to tell **policy-makers** about having [cardiac event/condition] or high cholesterol?<br><br>Is there anything else you would like to tell **health care providers** about having [cardiac event/condition] or high cholesterol?
**Recommendations to other people with high cholesterol**	Is there anything you would like to tell individuals who just got diagnosed with high cholesterol? What would you like them to know? How about people who recently were diagnosed with [cardiac event/condition]?
