# Supplementary material for: Experiences of People Diagnosed with High Levels of LDL Cholesterol and Atherosclerotic Cardiovascular Disease: Results from a Multinational Qualitative Study
Source: Glob Heart. 2025 Jul 15;20(1):63. doi: 10.5334/gh.1441 (PMC12273682; doi:10.5334/gh.1441)
Supplement: Supplementary Table 1. — Participant-described pathways to diagnosis. [file gh-20-1-1441-s3.pdf]

Supplementary Table 1. Participant-described pathways to diagnosis

Pathway	Illustrative quotes from interviewees	Frequency [n (%)]
Wellness check	**Participant from Australia who had not experienced an ASCVD hospitalization:** And my doctor, [doctor's name], who I've been seeing for over 25 years said, "I think you're at the age now where we should do a full bloods," and everything came back perfect except the cholesterol. – Participant from Australia who has not experienced an ASCVD event<br><br>**Participant from Brazil who had not experienced an ASCVD hospitalization:** The first time I came into contact with it was during a routine examination. At that time I was still in SUS (public health system), it was... at the health center and then the doctor asked for some routine tests.	25 (50%)
Visit for comorbidity	**Participant from Brazil who had not experienced an ASCVD hospitalization:** Yeah, actually because I've had diabetes for many years, 29 years, I've always had routine tests every 3-6 months, right? So the doctors were always asking me for tests, right? And then a routine cholesterol test showed altered cholesterol. And then the endocrinologist rushed to put me on medication, actually the endocrinologist didn't want to put me on medication.<br><br>**Participant from Australia who had not experienced an ASCVD hospitalization:** So, a while ago, I was... For a few years, I was taking tablets because I'd gone through a bit of a bipolar and depression period about 20 years ago and I was taking Epilim, which required checks for kidney function. And my doctor, [doctor's name], who I've been seeing for over 25 years said, "I think you're at the age now where we should do a full bloods," and everything came back perfect except the cholesterol.	12 (24%)
Visit for signs and symptoms	**Participant from Brazil who had not experienced an ASCVD hospitalization:** I had a lot of symptoms, a lot of discomfort, there were, there were a lot of things that made me go to the doctor at that time. Brazil, no event<br><br>**Participant from the United States who had not experienced an ASCVD hospitalization:** I started getting these symptoms. I know I've had migraines for a long time, so I've always had that but I was getting the fainting spells and I was getting the chronic fatigue. I was throwing up. I had trouble eating. [. . .] I had no exercise tolerance. I had trouble walking, weakness in my legs. I had a bunch of different odd symptoms that were happening to me and every time I would go to the doctor they would do bloodwork.	13 (26%)
