# Supplementary material for: Experiences of People Diagnosed with High Levels of LDL Cholesterol and Atherosclerotic Cardiovascular Disease: Results from a Multinational Qualitative Study
Source: Glob Heart. 2025 Jul 15;20(1):63. doi: 10.5334/gh.1441 (PMC12273682; doi:10.5334/gh.1441)
Supplement: Supplementary Table 2. — Participant-described attributes of an ideal treatment based on openended questions. [file gh-20-1-1441-s4.pdf]

Supplementary Table 2. Participant-described attributes of an ideal treatment based on open-ended questions

Attribute	Illustrative quotes from interviewees	Frequency [n (%)]
Satisfaction with current treatment	**Participant from Australia who had experienced an ASCVD hospitalization:** Well, basically my magic pill already is called [statin brand name]. I mean, it would be wonderful to be off medicine altogether but I'm not, so I'm very happy with the way it's been managed.	**16** (32%)
Non-daily dosing	**Participant from Brazil who experienced an ASCVD hospitalization:** I would like the treatment for cholesterol to be like one of those different contraceptives out there that you take, you take a quarterly dose and that's it. It's not just birth control, there's also vitamin D, there's vitamin, there's various vitamins that you take once every 3 months and you're, you know, you're immunized for those 3 months, I think that would be a way to take some of the worry off your mind, right?<br><br>**Participant from the United States who had not experienced an ASCVD event:** To be honest, it wouldn't be something that I would have to take every day. [. . .] I would – I, more than likely, would not miss a dose. [. . .] But that's the most important I can think of. Not missing a dose.	**21** (42%)
Non-capsule or non-tablet dosage form	**Participant from Brazil who had experienced an ASVCD hospitalization:** It would be injectable so I wouldn't have to take it every day, for example in the morning, when I take medicine in the morning it's horrible because I get nauseous a lot, when I take the medicine, I take it fasting so it's horrible, I would inject it, you inject it there, that's it, it's done.<br><br>**Participant from the United States who experienced an ASCVD hospitalization:** But also, throughout a couple of these questions, I've also thought if I could go in once a year for an infusion where I would only have to worry about it at that point in time as opposed to something that I'm taking on a day-in/day-out basis. I don't really want injections because those don't really work.	**16** (32%)
Minimal or reduced side effects	**Participant from the United States who experienced an ASCVD hospitalization:** It'd be fast. Side effects would be minimal. Something that you could do or take or have done that you would feel the difference immediately, almost like a recreational drug where you did it and you felt good. You thought this is something you should continue to do because you feel good and it's making you healthier and that sort of thing where you look better.<br><br>**Participant from Australia who had experienced an ASCVD hospitalization:** I would like a cholesterol tablet that didn't cause you cramps.	**11** (22%)

Improved efficacy	**Participant in Australia who experienced an ASCVD event hospitalization:** Your cholesterol decreases, rather than what I see it as keeping it more... it’s not rising or anything like that but it would be good if you took a pill and it just automatically decreased your cholesterol.<br><br>**Participant in the United States who experienced an ASCVD event hospitalization:** A perfect treatment would just bring all of my numbers in line with what is “considered normal.” I don’t think I can ask for anymore than that.	**10**<br><br>(20%)
Combination therapy for multiple conditions	**Participant in the United States who experienced an ASCVD event hospitalization:** About the medication I’m currently taking, okay. I would see if some of the pills could be rolled into one. [. . .]. Like I said, for blood pressure I’m taking three, then I take some for IBS, then my therapist has me on about three different meds. No, let’s just roll them all into one and let it do its job.	**6**<br><br>(12%)
Affordable	**Participant in the United States who had not experienced an ASCVD event hospitalization:** Probably making it free. It's not that expensive but still, over time, it's going to add up. Making medication free or very cheap and any medication that I need to go on further, moving forward, because I'm sure as I age, more medication might get changed or added. So, like yeah, make it more affordable, because seriously, I don't want to have to worry about when – my husband's our provider in this house, so when he can't be with me no more, who's going to pay for my medicine? I don't think Medicare's going to do it.	**2**<br><br>(4%)
